# Origin, Maturity Group and Seed Coat Color Influence Carotenoid and Chlorophyll Concentrations in Soybean Seeds

**DOI:** 10.3390/plants11070848

**Published:** 2022-03-23

**Authors:** Berhane Sibhatu Gebregziabher, Shengrui Zhang, Suprio Ghosh, Abdulwahab S. Shaibu, Muhammad Azam, Ahmed M. Abdelghany, Jie Qi, Kwadwo G. Agyenim-Boateng, Honey T. P. Htway, Yue Feng, Caiyou Ma, Yecheng Li, Jing Li, Bin Li, Lijuan Qiu, Junming Sun

**Affiliations:** 1 The National Engineering Research Center of Crop Molecular Breeding, Institute of Crop Sciences, Chinese Academy of Agricultural Sciences, 12 Zhongguancun South Street, Beijing 100081, China; berhane76@gmail.com (B.S.G.); zhangshengrui@caas.cn (S.Z.); fssuprio@gmail.com (S.G.); ashuaibu.agr@buk.edu.ng (A.S.S.); azaamuaf@gmail.com (M.A.); ahmed.abdelghany@agr.dmu.edu.eg (A.M.A.); qjycyz@gmail.com (J.Q.); k.g.agyenim.boateng@gmail.com (K.G.A.-B.); honeypusu@gmail.com (H.T.P.H.); 82101179104@caas.cn (Y.F.); mm-yoyo@hotmail.com (C.M.); 82101205019@caas.cn (Y.L.); lijing02@caas.cn (J.L.); 2 Crop Sciences Research Department, Mehoni Agricultural Research Center, Maichew 7020, Ethiopia; 3 Bangladesh Agricultural Research Institute, Gazipur 1701, Bangladesh; 4 Department of Agronomy, Bayero University, Kano 700001, Nigeria; 5 Crop Science Department, Faculty of Agriculture, Damanhour University, Damanhour 22516, Egypt; 6 MARA Key Laboratory of Soybean Biology (Beijing), Institute of Crop Sciences, Chinese Academy of Agricultural Sciences, 12 Zhongguancun South Street, Beijing 100081, China; 7 The National Key Facility for Crop Gene Resources and Genetic Improvement (NFCRI)/Key Laboratory of Germplasm and Biotechnology (MARA), Institute of Crop Sciences, Chinese Academy of Agricultural Sciences, Beijing 100081, China

**Keywords:** carotenoid, chlorophyll, germplasm origin, seed coat color, maturity group, regression analysis, soybean (*Glycine max* (L.) Merrill)

## Abstract

Soybean (*Glycine max* (L.) Merrill) seeds are abundant in physiologically active metabolites, including carotenoids and chlorophylls, and are used as an affordable source of functional foods that promote and maintain human health. The distribution and variation of soybean seed metabolites are influenced by plant genetic characteristics and environmental factors. Here, we investigated the effects of germplasm origin, genotype, seed coat color and maturity group (MG) on the concentration variation of carotenoid and chlorophyll components in 408 soybean germplasm accessions collected from China, Japan, the USA and Russia. The results showed that genotype, germplasm origin, seed color, and MG were significant variation sources of carotenoid and chlorophyll contents in soybean seeds. The total carotenoids showed about a 25-fold variation among the soybean germplasms, with an overall mean of 12.04 µg g^−1^. Russian soybeans yielded 1.3-fold higher total carotenoids compared with Chinese and Japanese soybeans. Similarly, the total chlorophylls were substantially increased in Russian soybeans compared to the others. Soybeans with black seed coat color contained abundant concentrations of carotenoids, with mainly lutein (19.98 µg g^−1^), β-carotene (0.64 µg g^−1^) and total carotenoids (21.04 µg g^−1^). Concentrations of lutein, total carotenoids and chlorophylls generally decreased in late MG soybeans. Overall, our results demonstrate that soybean is an excellent dietary source of carotenoids, which strongly depend on genetic factors, germplasm origin, MG and seed coat color. Thus, this study suggests that soybean breeders should consider these factors along with environmental factors in developing carotenoid-rich cultivars and related functional food resources.

## 1. Introduction

Carotenoids are lipophilic pigments that occur widely in nature and are distributed in plants, insects, fish, birds, algae, yeasts, archaea, fungi, bacteria and animals [1]. They are in the class of C_40_ isoprenoids comprising a large family with more than 750 members that are responsible for the red, yellow and orange colors of flowers, fruits and other plant organs [2]. Carotenoids can be categorized in to two groups based on their chemical composition: carotenes containing hydrocarbon only, and xanthophylls with one or more hydroxyl groups. Xanthophylls are oxygenated derivatives that include lutein, zeaxanthin, and β-cryptoxanthin, while carotenes include α-carotene, β-carotene, and lycopene. These components are the most abundant carotenoids in human blood plasma which account for more than 95% of the carotenoids found in human blood plasma [3].

Chlorophylls, fat-soluble plant pigments, comprise two major components: chlorophyll-a (chl-a) and chlorophyll-b (chl-b), which are green pigment photoreceptors found in all photosynthetic organisms. Carotenoids and chlorophylls are both chloroplast pigments involved in different functions, such as light harvesting, energy transfer, photochemical redox reaction, and photoprotection [4]. Carotenoids reflect a wide range of color pigments in plants and act as accessory pigments to chlorophylls in photosynthesis. Furthermore, both carotenoids and chlorophylls are well known for their nutritional and health benefits, involved in health promoting functions including anticarcinogenic, antioxidant and inti-inflammatory activities, as well as being used as food additives in various food industries, mainly due to their physico-chemical properties, and also the color that they impart to our food [5,6,7].

The vast majority of animals do not synthesize carotenoids de novo, and thus must obtain them through diet or partly modified through metabolic reaction [1]. Similarly, humans cannot synthesize carotenoids; instead, they ingest them in food or via supplementation. Humans get these health-promoting phytochemical compounds from different plant-derived food diets. Dark green leafy vegetables, colored fruits, root and tuber crops, cereals, legumes and unicellular microalgae are rich dietary sources of natural carotenoids [8]. It has been reported that legumes are rich sources of secondary metabolites (including carotenoids), well known for their potential benefits to human health [9,10].

The germplasm collections of soybean (*Glycine max* (L.) Merrill), which is among the globally staple legume crops, largely vary in their origins [11]. Soybean seed has been used as food and feed sources in many countries in the world and is an important part of traditional foods in many Asian countries due to its nutritional properties and functional characteristics. Nutritionally, soybeans are not only the primary source of protein and oil but also the world’s most important sources of secondary metabolites such as isoflavone, tocopherols, saponins, lipids and carotenoids, which are of strong therapeutic value [12,13].

The chemical composition of soybean seeds can be affected by genotype, planting location, environmental conditions, cultivation year, maturity group (MG) [14,15,16] and seed coat color [17]. Some studies have investigated the influence of geographical origins in various nutritional components (including isoflavones, protein, oil, fatty acids, tocopherols and folates) of soybean varieties [12,13,18,19,20,21]. Similarly, other recent studies evaluated soybean accessions of different origins and maturity groups for their nutritional quality attributes such as isoflavone [21], fatty acid compositions [22] and tocopherols [23] and found variation among genotypes and between MGs. Moreover, several soybean genotypes with different seed colors have previously been used for evaluating and improving seed chemical compositions such as isoflavone, fatty acids, carbohydrates, protein and oil [17]. Despite the fact that several research works have been carried out on the variability of soybean seed compositions, very little is known about soybean seed carotenoids and chlorophyll profile. To date, the contribution of soybean origin, MG and seed coat color to the carotenoid and chlorophyll contents of diverse soybean seed germplasms has not been reported so far. To our knowledge, only Monma et al. [24] analyzed the lipophilic pigment metabolites chl-a and -b as well as lutein and β-carotene of 50 Japanese soybean varieties with various seed colors and different maturity stages.

Though carotenoids in soybean seeds have not been given more attention in breeding research programs, increasing carotenoid content in soybean seeds is believed to be an effective way to improve the nutritional value of soybean derived foods. The evaluation of unexploited soybean germplasms on a large scale with diversified phytochemical properties helps to obtain elite accessions for the sustainable improvement of seed carotenoid accumulation in soybean breeding programs. High genetic diversity provides an opportunity for plant breeders to investigate accurate and effective strategies for improving the desired traits of soybeans [25]. In an attempt to achieve this strategy, in this study, 408 diverse soybean germplasm accessions of different origins (China, Japan, Russia and the USA), MGs and various seed coat colors were evaluated under the same environmental conditions. An understanding of the carotenoid and chlorophyll fluctuations in different soybean origins with various MGs and seed color would provide a more complete characterization of the nutritional compositions for the final utilization of soy products in different food, pharmaceutical, nutraceutical and cosmetic industries. Thus, we hypothesized that the aforementioned factors had a significant effect on soybean seed carotenoid and chlorophyll compositions. The present study was undertaken to (i) comprehensively analyze the variation in carotenoid and chlorophyll concentrations in large panel soybean accessions of diverse origin, (ii) investigate the influences of seed coat color and MG on soybean seed carotenoid and chlorophyll profile and concentrations and (iii) identify elite soybean accessions with a substantial concentration of carotenoids across environments. The derived information will help breeders and producers to develop and disseminate breeding strategies for enhancing carotenoid concentration in soybean seeds.

## 2. Results and Discussion

### 2.1. Comprehensive Natural Variation of Seed Carotenoid and Chlorophyll Contents in Soybean

The concentration of major carotenoids such as lutein, zeaxanthin, and β-carotene, as well as chlorophyll components, including chl-a and -b, were quantified (Table 1). Significant variations (*p* < 0.001) in carotenoids and chlorophylls were observed among the soybean accessions, showing the existence of wide genetic differences and thereby a good opportunity to obtain valuable genetic resources for soybean breeding (Table S3). The overall means and variations of the traits across two planting years are summarized in Table 1. The total carotenoid content ranged from 1.35 to 33.09 µg g^−1^ with an overall mean of 12.04 µg g^−1^, showing a comparative advantage compared with previous studies reporting average total concentrations of 6.32 µg g^−1^ [24]. The highest and lowest contents were obtained from ZDD11183 and ZDD25115 Chinese accessions, respectively (Table S1), with about a 25-fold variation between the germplasms for total carotenoid content. 

Individual carotenoid components were also significantly influenced (*p* < 0.001) by the genotypic effect. Lutein ranged substantially from 1.35 to 32.08 µg g^−1^, with the highest and lowest concentrations obtained from the above-mentioned soybean accessions, respectively. Notably, lutein was the most abundant component, which is in line with several reports showing that lutein is the dominant component of carotenoids in many legume crops [24,26,27]. Surprisingly, in the present study, it covered 97.8% of total carotenoids, and obtained from all tested accessions, which is consistent with the previous report showing 96.6% lutein coverage [28]. Previous studies documented that some soybean inbred lines have been developed to enhance soybean seed lutein content [29,30]. Importantly, this study provides new insights to help obtain more parental lines so as to generate new breeding lines for soybean lutein content improvement at a global level. Likewise, it is interesting to note that lutein is relatively found to be the most stable trait compared with the coefficient variation (CV) value of the others, suggested to be due to strong genetic control that boosts their performance even when grown under diverse field conditions. Concerning β-carotene, soybean accessions accumulated a mean concentration of 0.52 µg g^−1^ β-carotene, with the maximum (2.29 µg g^−^^1^) and minimum (0.04 µg g^−1^) values unequivocally observed in WDD02873 from Russia and ZDD14267 from China, respectively, substantially 8.66-fold higher than the previous findings [28], which could additively promote total antioxidant activity in soybeans [31]. Collectively, our study identified outstanding soybean accessions with the higher accumulation of carotenoid components compared to previous studies, most probably due to the presence of large germplasm collections in our study, which helped to explicitly analyze the genetic variability. Notably, our results suggest that utilizing genetic resources with abundant genetic differentiation helps to increase the contents of economically desired traits in soybeans, which is supported by previous studies on genetically diversified chickpea and pea accessions [32].

In the present study, the analysis of variance showed that total chlorophyll content varied significantly (*p* < 0.001) (Table S3) and was found in the range of 0.36–87.68 µg g^−1^ with a mean of 4.05 µg g^−1^ (Table 1). Among the soybean germplasms, the ZDD06375 accession from China contained the highest level of total chlorophyll, whereas the ZDD02764 accession from China had the lowest total chlorophyll content. Similarly, the concentrations of chl-a and-b were highly significantly influenced (*p* < 0.001) due to the genetic variability of the soybean accessions. Chl-a and -b were obtained from 57% and 96% of the total accessions, respectively, implying that the seeds of all soybean accessions could not contain chlorophylls. Soybean seeds contained a mean of 5.06 µg g^−1^ and 1.58 µg g^−1^ chl-a and chl-b, respectively (Table 1), confirming that chl-a exceeds chl-b approximately by a 3:1 margin [6]. Some studies have detected the lipophilic pigment metabolites chl-a and-b as well as lutein and β-carotene [31,33], but no clear quantified data of the chlorophylls were included in the reports. Thus, the present study explicitly indicated that chlorophyll contents may vary depending on soybean seeds of different genetic diversities.

It was observed that cultivation year caused significant variation (*p* < 0.001) in the contents of carotenoids and chlorophylls of soybean seeds, indicating that breeders should take not only the genotypic effect but also seasonal variations into consideration during soybean seed lipophilic pigments production. Several studies have confirmed the effect of planting season on soybean seed compositions such as carotenoids, isoflavone, amino acids, oil and fatty acids, among others [14,21]. In the present research, the interaction of accession by year had no significant effect (*p* > 0.05) (Table S3), indicating that genetic factor plays a major role in the accumulation of carotenoids and chlorophylls in various soybean seed germplasm accessions.

### 2.2. Germplasm Origin Differently Affected Soybean Seed Carotenoid and Chlorophyll Concentrations

**Figure 1:**
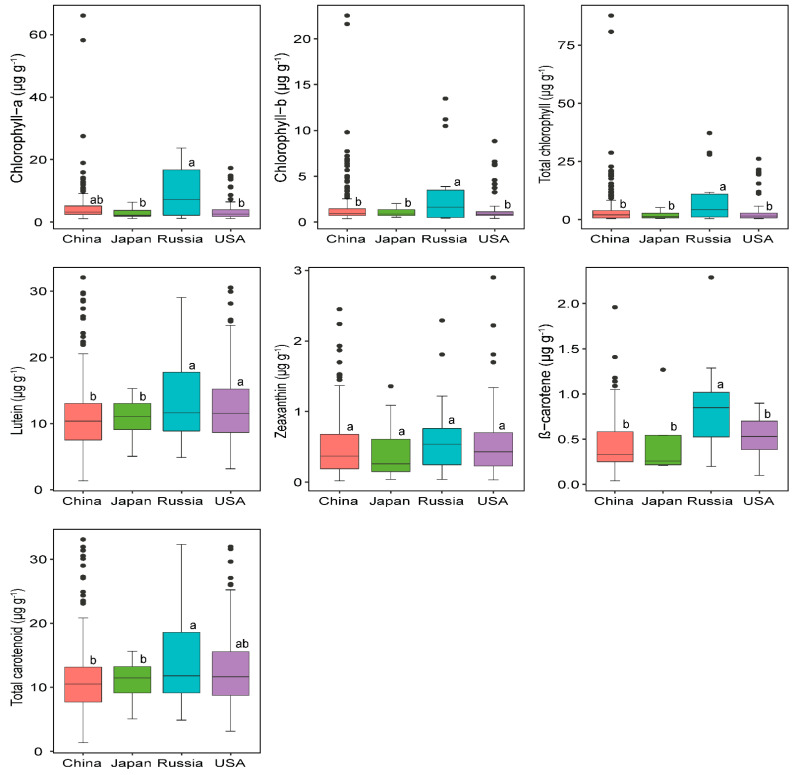
Variations in carotenoid and chlorophyll contents among soybean germplasm accessions originated from China (*n* = 236), USA (*n* = 135), Russia (*n* = 19) and Japan (*n* = 18). *n* represents number of soybean accessions. The lines across each box plots indicate the medians. Different lower-case letters (a, and b) indicate statistically significant difference at *p* < 0.05 level among the germplasm origins.

In the current study, carotenoid and chlorophyll contents significantly varied by germplasm origin (Table S3), which is consistent with previous studies that reported on other soybean bioactive compounds [12,19,34]. The variation in carotenoid and chlorophyll contents among the four germplasm origins is shown in Figure 1. The total carotenoid level was significantly higher in accessions originated from Russia (14.78 µg g^−1^) and the USA (12.58 µg g^−1^), whereas the lowest mean content was observed in Japanese (11.10 µg g^−1^) and Chinese (11.41 µg g^−1^) accessions, which shows the existence of wider variability among the soybean germplasms, which could be attributed to environmental and genetic factors.

Interestingly, the same trend was followed in the lutein content, where the optimum quantity of lutein was obtained from Russian and USA accessions. Our results also highlighted that Russian soybean accessions contained a significantly superior concentration of β-carotene, while the others showed statistically similar responses. Growing year is another equally important factor that impacts soybean seed metabolite content, as reported by Ashokkumar et al. [26] on the carotenoid contents of pea and chickpea. In our study, lutein and total carotenoid contents were significantly influenced (*p* < 0.001) by the growing year, whereas zeaxanthin was significantly affected neither by the country of origin nor growing year, showing that its concentration is similar across the cropping years, irrespective of germplasm origin (Table S3), implying that zeaxanthin was relatively stable in soybean accessions. 

The analysis of variance (ANOVA) showed that chlorophyll components were significantly influenced (*p* < 0.05) by the geographical origin of accessions (Table S3). Russian accessions followed by Chinese accessions contained significantly higher chlorophyll concentrations, while USA and Japanese accessions contained relatively lower concentrations (Figure 1), attributed to the genetic variability of the accessions to perform in different environmental conditions, corresponding to the study of Song et al. [35], who found that accessions from different geographical origins (such as Japanese and Chinese) are genetically distinct, resulting in various biochemical contents in soybean. In summary, understanding the origin of the germplasm and cultivation year is crucial in soybean breeding strategies to improve carotenoid contents, as suggested by several other studies on other desirable soybean seed nutritional compositions [18,20,21,22].

### 2.3. Seed Coat Colors Differently Affected Soybean Seed Carotenoid and Chlorophyll Concentrations

**Figure 2:**
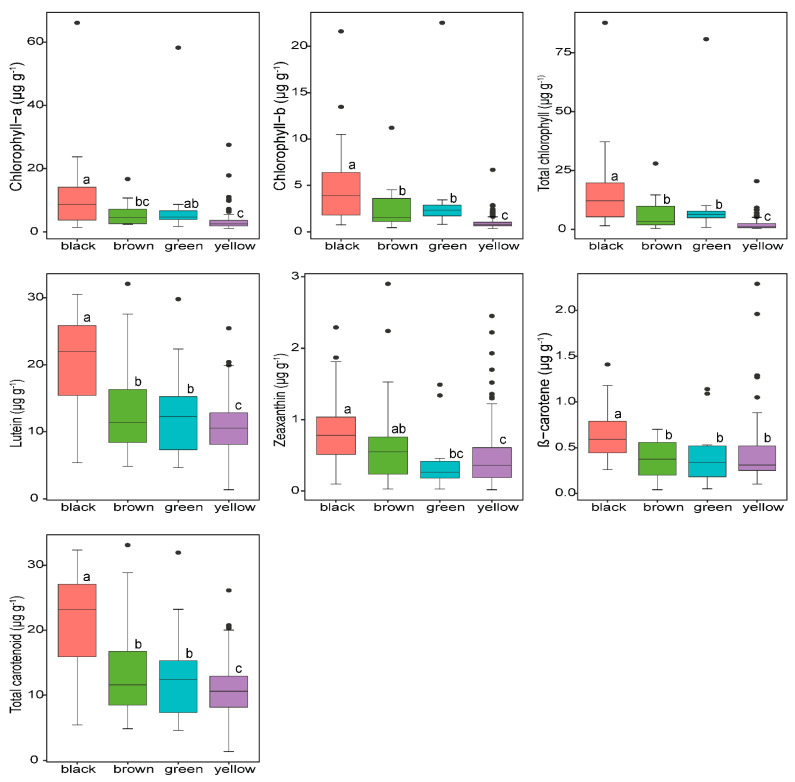
Seed carotenoid and chlorophyll concentrations of soybean accessions with different seed coat colors: black (*n* = 41), brown (*n* = 18), green (*n* = 18) and yellow (*n* = 331). *n* represents sample size. Different lower-case letters (a, b, c) indicate statistically significant difference at *p* < 0.001 level among the seed coat colors.

Soybeans exhibit natural variation in seed coat colors that impact the nutritional composition of soybeans. Soybean seeds tested in our experiment were available in the form of yellow, black, green, and brown seed colors (Figure S1), formed due to the accumulation of different pigment-stimulating metabolites [36,37]. The results showed that the seed color differences contributed differentially to the variations in the contents of carotenoid and chlorophyll components (Table S3), indicating that seed coat color should be taken as an important factor in soybean seed nutritional components. As shown in Figure 2, the highest average total carotenoid content was observed in black seeds (21.04 µg g^−1^), followed by brown (13.93 µg g^−1^) and green soybeans (13.15 µg g^−1^), while yellow seeds contained about 1.97-fold lower total carotenoid content than that of black seeds. A similar trend was also followed in lutein among the soybean seed colors, where significantly superior lutein content was found in black soybean seeds, which suggests that accessions with a black seed coat color can be preferentially selected by breeders to develop lutein-rich elite cultivars. The results were coherent with earlier studies [9,34,38,39] that indicated black soybeans are rich sources of phytochemicals, including carotenoids, anthocyanins, tocopherols and isoflavones. The presence of more carotenoids in black soybeans could be most probably due to relatively smaller seeds (Figure S2), which is consistent with previous studies on soybean seed weight which found that smaller seeds contained higher contents of targeted metabolites [12,29,40]. In other legumes, Ashokkumar et al. [32] also found considerably high contents of carotenoids in black cultivars as compared to their yellow counterparts.

Zeaxanthin, stereoisomers of lutein specifically present in the macula and lens of the human eye, showed significant differences among the seed colors, with the highest mean concentration observed in black seeds (0.85 µg g^−1^), which, however, was statistically similar to brown colored seeds. Though the majority of soybeans in our experiment as well as globally [41] exhibit yellow seed coats, they had the lowest mean zeaxanthin content (0.44 µg g^−1^) (Figure 2). Concerning β-carotene, accessions with black seed coat colors had approximately 1.5-fold higher β-carotene content than the others, showing that soybeans with black seed colors are rich in β-carotene, thereby resulting high photoprotective and antioxidant capacities in soybeans, which is in agreement with previous reports [42,43].

The seed coat color of soybeans also highly significantly (*p* < 0.001) influenced the concentrations of chlorophyll (Figure 2 and Table S3). By comparison, soybeans with black seed colors contained considerably higher contents of total chlorophyll than accessions with green, yellow and brown seed colors, suggesting that differently colored seeds can possess various types of bioactive and other beneficial components [24]. As shown in Figure 2, the mean concentrations of total chlorophyll in black seed colors were 8.1 times higher than yellow-colored accessions (1.87 µg g^−^^1^), which statistically had the lowest mean concentration. In the case of individual chlorophylls, the same trend was observed in chl-b; however, black and green seed coat colors responded similarly in chl-a. Recently, Jo et al. [44] reported that soybean germplasms with black seed coat and green cotyledon are rich in chlorophylls and other functional nutrients. Likewise, several studies have documented that black soybeans have been used as traditional ingredients in medicinal treatments (folk medicine) in Asian countries including China, Japan and Korea due to their content of potentially active phytochemicals in their seed coat [36,45].

Several factors, including planting season, can affect the soybean seed metabolites. As shown in Table S3, cultivation year had a pronounced effect on the contents of soybean chl-b and carotenoid components except β-carotene, possibly due to climatic conditions, as reported by Ashokkumar et al. [26] in pea and chickpea carotenoid profile, who found 1.18-fold chickpea lutein and 1.07-fold total carotenoid concentration variation between two planting years. The year by seed coat color interaction also had a significant impact on chl-b and zeaxanthin concentrations, suggesting seasonal variation over the years can affect chlorophyll and carotenoid components of soybeans of different seed colors. Taken together, our results suggested that black soybeans are reliable resources for producing more lipophilic pigments, particularly carotenoids, which can be used in food additives and medicinal treatments to enhance human nutrition and health. Remarkably, a high level of carotenoids contributes to the greater functional food source and pharmacological capacities of black soybeans than other colored soybeans. In addition, our study demonstrates that seed coat color is among the numerous qualitative characteristics of soybean seed that determines the biochemical composition of soybeans; thereby, this information is helpful for breeders to consider this agronomic trait during carotenoid-rich cultivar development so as to enhance the functional and nutritional values of soybeans.

### 2.4. The Maturity Groups Differently Affected Soybean Seed Carotenoid and Chlorophyll Concentrations

**Figure 3:**
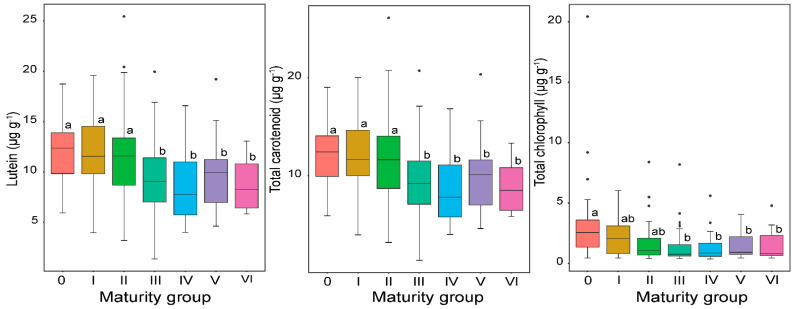
Seed carotenoid and chlorophyll concentrations of yellow-seed-coat-colored soybean accessions of different maturity groups. Different lower-case letters (a, b and ab) indicate statistically significant difference at *p* < 0.001 level among the maturity groups. Number of accessions in MG 0 = 61, MG I = 57, MG II = 57, MG III = 80, MG IV = 37, MG V = 24 and MG VI = 15. MG represents maturity group.

Soybean accessions are classified into different MGs based on their photoperiod (day length) requirements. Maturity group is among the major agronomic characteristics that determine soybean seed quality. In the present study, the ANOVA revealed that MGs contributed differently to the carotenoid and chlorophyll responses in soybean seeds (Table S3), and is supported by several studies reporting the effect of MG differences on soybean seed quality characteristics [16,21,22,46]. Figure 3 shows the average contents of carotenoid and chlorophyll components in soybean accessions on the basis of MG. The early MGs (MG 0–MG II) contained the highest mean total carotenoid concentrations, while the other MGs, which were significantly similar, generated lower mean contents of total carotenoids. The same pattern was also observed in lutein, where a decreasing trend of lutein towards late MGs was observed, implying that early matured soybeans accumulate more lutein, which might be attributed to climatic conditions and lutein accumulation pattern in short-season soybean genotypes, which are naturally originated and distributed in high-latitude regions. This concurs with the study of Ghosh et al. [23], who reported that early MG soybean accessions, originated and distributed in high-latitude regions, contained high levels of soybean seed nutritional compositions, including tocopherols.

The total chlorophyll was highly significantly accumulated in the earliest soybean MGs, while soybean accessions corresponding to the MG from MG III to MG VI exhibited lower total chlorophyll concentrations (Figure 3). As shown in Figure S3, individual chlorophylls, zeaxanthin and β-carotene contents did not show significant responses to MGs, indicating that these components are not more sensitive to the day of maturity. Most importantly, alongside the effects of MGs on the biochemical responses of the targeted pigments, cultivation year had a significant contribution to the contents of carotenoids and chlorophylls (Table S3), implying that seasonal changes can influence the carotenoid and chlorophyll concentrations and the profile of soybeans of different MGs, which is supported by similar reports on soybean isoflavone [16] and tocopherol [23]. Additionally, the MG by year interaction had significant effect (*p* < 0.001) on lutein, total carotenoids and chlorophylls, which is in consonance with the earlier study on soybean seed nutritional characteristics [21]. The significant effect of year by MG interaction on the aforementioned components underlines the high sensitivity of soybeans to the growing seasonal climatic variations, including photoperiod and temperature, during seed maturation [47].

As no studies have been carried out so far on the effect of soybean MGs on carotenoid and chlorophyll contents, interestingly, the present study pointed out that the variation of these bioactive compounds depended not only on the effect of genotype and environment factors, but also on the effect of MG, suggesting that breeders should take into account MGs by themselves beyond to the genotype effect during soybean production. Overall, understanding the differential responses of the components to MGs is magnificently important to harmonize soybean cultivar with its best regional adaptation and further plays a significant role in the breeding and production of quality trait soybeans in world geographical regions.

### 2.5. Principal Component Analysis Based on Origin and Seed Coat Colors

**Figure 4:**
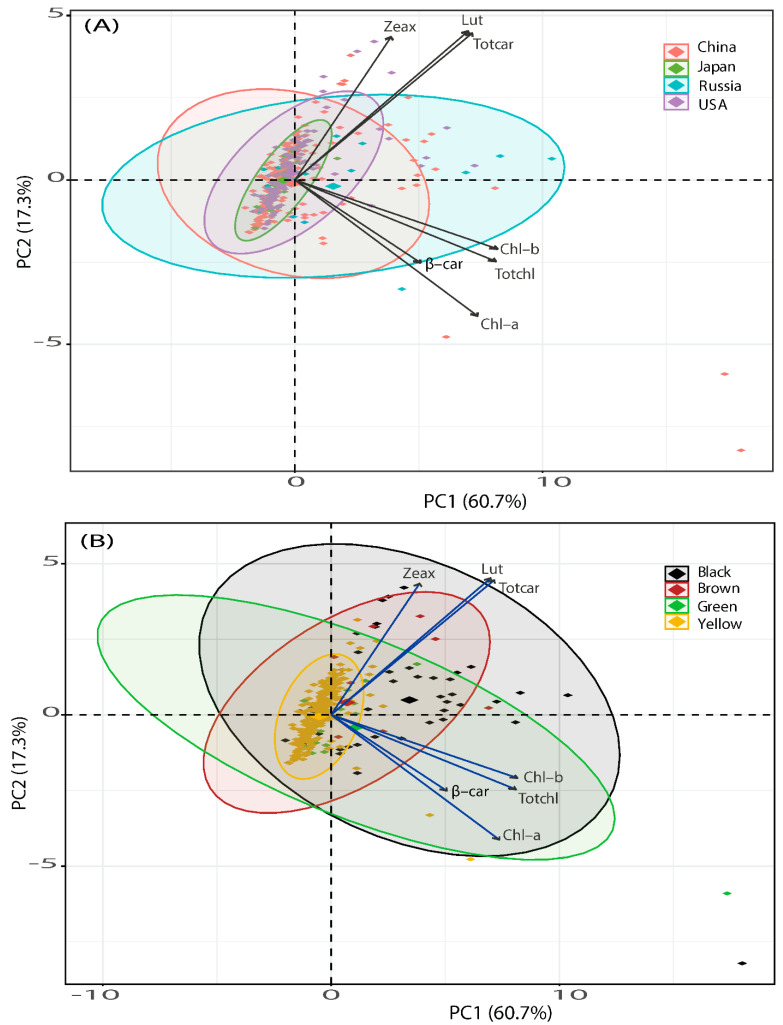
Principal component analysis (PCA) on carotenoid and chlorophyll components of world soybean accessions based on (**A**) country of origins; (**B**) seed coat colors. Each of the points on the biplots with different colors and symbols represents a single soybean accession signifying origin and seed coat color. Lut, lutein; Zeax, zeaxanthin; β-car, β-carotene; Totcar, total carotenoids; Chl-a, chlorophyll-a; Chl-b, chlorophyll-b; Totchl, total chlorophylls.

In this study, principal component analysis (PCA) was carried out to assess the variation of carotenoid concentration and profile of soybean accessions based on various seed coat colors and germplasm origins. Figure 4A,B show the PCA, outlining the carotenoid and chlorophyll concentrations and profile differences among soybeans of different origins and seed coat colors, respectively. In Figure 4A, carotenoids and chlorophylls data yielded two principal components (PCs), which accounted for 78% of the total variances and were positively loaded with every component of carotenoids and chlorophylls. The first component of the PCA (PC1) accounted for 60.7% of the data set variation, and the second component (PC2) explained an additional 17.3% of the observed variation, indicating that variations in the components of carotenoids and chlorophylls were observed based on the country of origins.

In Figure 4A, it can be seen that chl-b (20.39%) contributed the highest to the variance in PC1, followed by total chlorophyll (20.06%) and chl-a (16.58%), while total carotenoid (21.35%) preceded by lutein (21.98%) were the best contributors to the total variance in PC2. As shown in Figure 4A, a large number of soybean accessions of USA origins were densely scattered close to the carotenoid components (lutein, zeaxanthin and total carotenoid), signifying that these accessions tend to contain high levels of individual and total carotenoids across multiple environments, confirming to the results in Figure 1. On the other hand, Chinese soybeans followed by Russian soybeans were largely distributed around the chlorophyll components, suggesting that soybeans of these origins are rich sources of chlorophylls, despite the quantitative significant differences exhibited among them.

The PCA of carotenoids and chlorophylls for the seed coat color differences is presented in Figure 4B, where the score plots in PC1 and PC2 illustrate reasonable clustering appearance according to the differences in seed coat colors. The PCA, which accounted collectively for 78% of total variance, unraveled the existence of differences in carotenoid and chlorophyll concentrations of various seed coat colors. Though the number of black soybeans tested (41) was lower compared to yellow ones (331), they were more diverse around the carotenoid components (mainly lutein and total carotenoid), while the others were sparsely distributed, signifying that black soybean accessions are rich sources of these components. Similarly, comparatively more diverse black soybeans were observed around β-carotene and chlorophyll components, indicating that accessions with these seed coat colors tend to preserve high levels of β-carotene, individual and total chlorophylls, which is in agreement with the results in Figure 2. The contribution of variables followed the same trend as shown in Figure 4A, where the variation in PC1 was largely attributable to chl-b (20.39%), followed by total chlorophyll (20.06%) and chl-a (16.58%), while lutein (21.98%) and total carotenoid (21.35%) followed by zeaxanthin (20.27%) were the best contributors to the total variance in PC2.

The PCA reveals variations and associations among parameters and identifies major contributing variables, as reported by Ramadan et al. [48] Here, all the carotenoid and chlorophyll components positively contributed to the total variance and showed positive associations, which are attributed to common functions including light-harvesting, energy transfer to the photosynthetic reaction center, photochemical redox reactions and photoprotection [4], as well as antioxidant activity [49]. The positive associations among individual components observed here were also previously reported in chickpeas and peas [32].

### 2.6. Regression Analyses to Seed Coat Color and 100-Seed Weight

**Figure 5:**
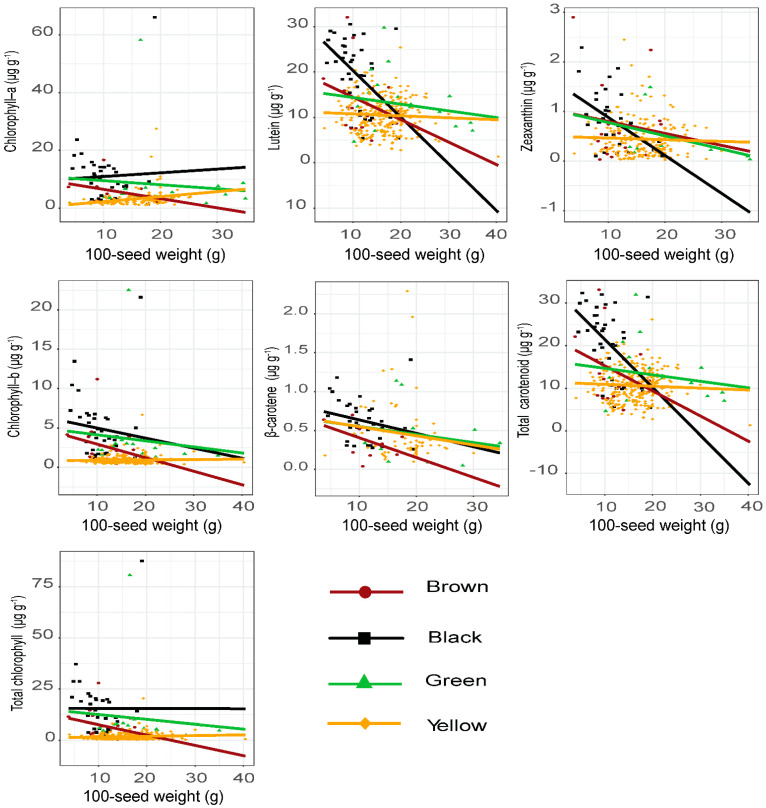
Linear regression analyses between the lipophilic pigment components and 100-seed weight in brown (*n* = 18), black (*n* = 41), green (*n* = 18) and yellow (*n* = 331) soybean germplasm accessions.

The linear regression relationships of carotenoid as well as chlorophyll components in various colored soybeans in relation to 100-seed weight are shown in Figure 5. The concentration of carotenoid components (lutein, zeaxanthin, β-carotene and total carotenoids) increased with the decrease in 100-seed weight in black, brown and green seed coat colors (Figure 5 and Table S4). An extremely weak relationship between 100-seed weight and the components of carotenoids was observed in yellow soybean seeds, indicating that seed weight did not make a significant contribution to the response of carotenoids in yellow-colored soybeans. A previous study carried out regression analyses based on seed coat color and found strong linear relationships with different responses of soybean seed metabolites relative to 100-seed weight [17]. In the present study, collectively, the contents of carotenoid components increased significantly with the decrease in 100-seed weight in black-colored soybeans, implying that smaller soybean seeds accumulate more carotenoids, which is in line with the study of Kanamaru et al. [29]. Moreover, Abbo et al. [50] found that seed weight was negatively associated with components of carotenoids, such as lutein, zeaxanthin and β-carotene in chickpea seeds. Furthermore, previous studies found higher contents of total isoflavone and phenolic compounds in low-seed-weight soybeans [12,40]. Notably, this study can suggest that seed weight plays a significant role in the synthesis and accumulation of carotenoids in soybean seeds of various seed coat colors. Taking into account the demand of large-seeded soybeans under commercial breeding programs by consumers, here, we recommend further advanced physiological and genetic studies to improve seed weight without affecting the levels of carotenoid concentrations.

Similar to carotenoids, the same trend was followed in chlorophylls, where the levels of total chlorophylls increased with the decrease in 100-seed weight in black, green and brown soybean seeds, while inverse response to 100-seed weight in yellow seeds (Figure 5 and Table S4). Briefly, the inverse relationship between chlorophylls and seed weight could be most probably due to the presence of pleiotropic effects that can hamper the synergistic development of 100-seed weight and carotenoids as well as chlorophylls. Overall, carotenoid and chlorophyll components exhibit diverged responses in relation to seed coat color and seeds’ weight within the soybean germplasm accessions. Several studies documented that the concentration of metabolites had significant variations in soybeans with different 100-seed weights [12,36,40].

### 2.7. Soybean Accessions with Prominent Content of Lutein and Total Carotenoids

Soybeans showed a wide range of variations in their lipophilic pigment contents. The variability of soybean seed lutein content has been analyzed [24,33]. Previous findings showed that soybean genotypes grown in Maryland [51] and Chinese soybeans grown in northeast China [30] produced 27.20 µg g^−1^ and 23.96 µg g^−1^ lutein, respectively. Our results identified ten soybean accessions that contained a lutein concentration higher than 27 µg g^−1^ (Table 2), and we suggested that these should be used as parents in soybean breeding. The identification of high-lutein soybean germplasm accessions is important for breeding high-quality soybeans.

Table 2 also shows soybeans that contain substantial concentrations of total carotenoids. We identified ten elite soybean accessions with total carotenoid contents greater than ≈29 µg g^−1^, of which seven of them had black, one green and two brown seed coats, which can help consumers adjust their preferences for soybean seed colors, and we suggested that these should be used in daily food resources for promoting and sustaining health functions. It is interesting that all the selected accessions contained substantial concentrations of lutein, zeaxanthin and β-carotene components. Both lutein and zeaxanthin, the xanthophyll carotenoids accumulated in the macula and lens, are the key carotenoid components responsible to prevent eye diseases, mainly associated with reducing the risk of age-related macular degeneration and cataract [52]. Collectively, these promising germplasms with unique carotenoid profiles will be considered as potential donors of this important nutritional and quality trait and suggested to be used as sources of genetic materials in conventional and/or molecular breeding purposes, including biofortification programs so as to enhance grain quality, thereby the supply of natural ingredients for food products of modern food industries.

## 3. Materials and Methods

### 3.1. Plant Materials and Field Experiments

This study employed natural populations consisting of 408 diverse soybean accessions (Table S1), originated from China (236 accessions), the USA (135 accessions), Russia (19 accessions) and Japan (18 accessions). These germplasms were received from the Chinese National Soybean Gene Bank (CNSGB), Institute of Crop Sciences, Chinese Academy of Agricultural Sciences. The CNSGB conserves 23,587 Chinese germplasm accessions collected from the whole of China, mainly the northern, Huang Huai Hai Valley and southern regions [53]. The genotypes included in this experiment were also classified into yellow (331 accessions), black (41 accessions), green (18 accessions) and brown (18 accessions) seed coat colors (Table S1 and Figure S1). In addition, seven MGs, including MG 0 (61 accessions), MG I (57 accessions), MG II (57 accessions), MG III (80 accessions), MG IV (37 accessions), MG V (24 accessions) and MG VI (15 accessions) were formed (Table S1). Here, to avoid the interferences of various seed-coat-colored soybeans with differential responses to lipophilic pigments, the same seed-coat-colored accessions (only yellow) were taken to explicitly investigate the effects of MGs on concentrations of carotenoids and chlorophylls in soybean seeds.

The genotypes were planted over two years (2017–2018) in Changping (40°13′ N, 116°12′ E), Beijing and Sanya (18°24′ N, 109°5′ E), Hainan province. The experiments were conducted each year following sowing from the middle of June in Changping and from the middle of November in Sanya. The experiment was laid out in a randomized, incomplete block design with the two planting locations deployed as replications. The experimental unit was a row with a width of 3 m accompanied with 0.5 m and 0.1 m inter and intra row spacing, respectively. Each row contained 20 plants as a seed source that was used for subsequent carotenoid and chlorophyll content measurements. Nitrogen, phosphorus and potassium fertilizers were applied at the rate of 30, 40 and 60 kg ha^−1^, respectively. Other recommended agronomic practices were previously reported [22]. The mean monthly temperature, rain fall and sunshine in the experimental locations are shown in Table S2.

### 3.2. Chemicals and Reagents

The carotenoid standards, such as zeaxanthin (CAS: 144-68-3, purity ≥ 85%), β-carotene (CAS: 7235-40-7, purity ≥ 98%), and chlorophyll standards, including chlorophyll-a (CAS: 479-61-8, purity ≥ 85%) and chlorophyll-b (CAS: 519-62-0, purity ≥ 90%), were purchased from Shanghai Yuanya Biotechnology Co., Ltd. (Shanghai, China), while the carotenoid standard lutein (CAS:127-40-2, purity ≥ 96%) was purchased from Sigma-Aldrich (St. Louis, MO, USA). HPLC analytical-grade methanol, acetone, ethanol, and ammonium acetate were purchased from Thermo Fisher Scientific Co., Ltd. (Fair Lawn, NJ, USA). Methyl *tert*-butyl ether and butylated hydroxytoluene, HPLC-grade chemicals, were purchased from Mreda Technology Inc., USA and Shanghai Macklin Biochemical Co., Ltd., Shanghai, China, respectively. Ultrapure water (Milli-Q) was obtained from a Millipore system (Millipore, Billerica, MA, USA).

### 3.3. Extraction and Determination of Carotenoids and Chlorophylls

The detailed procedure used for extracting and analyzing carotenoid and chlorophyll compositions of matured soybean seeds has been recently reported [42]. In brief, fine powder was obtained from 20 g of seeds of each soybean accession grinded with a sample preparation Mill (Retsch ZM100, Φ = 1.0 mm, Rheinische, Germany). After grinding, 100 mg of powder from each sample was accurately weighed out using an electronic analytical balance (Sartorius BS124S, Gottingen, Germany), and placed in a 2 mL micro-centrifuge tube preloaded with 1.5 mL of a mixture of ethanol and acetone solvents at a 1:1 ratio. The 0.1% butylated hydroxytoluene (*w*/*v*) was added to the solvents to keep carotenoids and chlorophylls stable. The mixture was placed and shaken in an ultra-sonic water bath (Ningbo Scientz Biotechnology Company Ltd., Ningbo, China) for 20 min at room temperature. Supernatant was collected via centrifugation at 13,000 rpm for 10 min at 4 °C and transferred to a new centrifuge tube for another centrifugation at 13,000 rpm for 5 min at 4 °C. The collected supernatant was then filtered using a 0.2 µm pore dimension YMC duo-filter (YMC Co., Kyoto, Japan) with the help of a sterile syringe (Jiangsu Zhiyu Medical equipment Co., Ltd., Jiangsu, China) and placed in a 1.5 mL amber glass HPLC vial (AS ONE, Ningbo, China) for subsequent analysis. The carotenoid and chlorophyll extracts were analyzed using an Agilent 1100 Model HPLC instrument (Agilent Technologies, Santa Clara, CA, USA) equipped with a Hewlett-Packard Model 1050 solvent delivery system and a reverse-phase C30 YMC Carotenoid (250 × 4.6 mm I.D., S-5 µm, YMC CO., Kyoto, Japan) column coupled with a UV-Vis detector (Santa Clara, CA, USA) set at a wavelength of 450 nm. Gradient elution was performed with mobile phases consisting of methyl *tert*.-butyl ether, methanol-10 mM ammonium acetate and ultrapure water, delivered at flow rate of 0.9 mL min^−1^ with an injection volume of 20 µL. Finally, the concentrations of each component were calculated using the formula [54]: carotenoid or chlorophyll (μg g^−1^) = [C*x* (μg mL^−1^) * V (mL) * D]/Wt (g); where, C*x* = the concentration of each component calculated from the standard calibration curve, V = volume of the extracting solvent, D = any dilution factor, and Wt = sample weight in dry bases. The total carotenoid and total chlorophyll concentrations were described as the sum of individual carotenoid and chlorophyll components, respectively.

### 3.4. Statistical Analysis

The combined data were subjected to ANOVA using the procedure of general linear model (PROC GLM) (SAS version 9.1, SAS Institute Inc., Cary, NC, USA) to determine the effects of accessions, germplasm origin, seed coat color and MG on variability of carotenoid and chlorophyll concentrations. Accession, germplasm origin, MG and seed color were considered as fixed effects, while locations together with years were set as random effects. Differences were considered statistically significant at *p* < 0.05. Multiple comparisons of means were performed using Tukey’s honestly significant difference (HSD) test. Boxplots were drawn to show the distribution and variation of seed carotenoid and chlorophyll compositions among the four countries of origin, seven MGs as well as four seed coat color types. Principal component analysis (PCA) was performed to identify components with high discriminatory properties, which in turn were used to group accessions based on their origin and seed coat color as well as to show the contribution of each component to the total variation among the countries of origin and seed color types. Regression analysis was performed to establish the relationship between 100-seed weight and lipophilic components within the corresponding seed coat colors. The PCA, regression analysis and boxplots were analyzed using R statistical software version 3.6.3 (R Foundation for Statistical Computing, Vienna, Austria).

## 4. Conclusions

In this study, the variability in concentrations of carotenoids and chlorophylls across soybeans of diversified origin along with various seed coat colors and MGs were investigated. The results showed that carotenoids and chlorophylls varied in terms of countries of origin, genotype, seed coat color and MGs. Wide variation existed among individual and total carotenoids as well as chlorophyll levels in soybeans. Chinese and Japanese soybeans contained lower total carotenoids, while Russian and USA soybeans produced significantly similar high contents. The higher total chlorophylls were largely observed in Russian, followed by Chinese. In terms of seed coat colors, black soybeans contained significantly abundant concentrations of carotenoids and chlorophylls, implying that black soybeans are rich sources of lipophilic pigments. Higher carotenoid and chlorophyll concentrations were significantly presented in early- rather than late-maturing soybeans, showing that MG should be considered as an influential factor in soybean seed compositions. Altogether, this result demonstrates that germplasm origin, seed coat color, MG and 100-seed weight differently affected the concentrations of targeted carotenoid and chlorophyll components in soybean seeds. Understanding the profile of carotenoids and chlorophylls in soybeans and their responses to germplasm origin, seed coat color and MGs is necessary to better improve the quality and bioactive constituents of soybean seeds and ultimately provide valuable information to modern food industries developing nutrient dense foods.

## Figures and Tables

**Table 1:** Descriptive statistics for the concentrations of carotenoid and chlorophyll traits in soybean accessions grown in two locations for two years.

Traits	Min (µg g^−1^)	Max (µg g^−1^)	Range (µg g^−1^)	Mean (µg g^−1^)	SD	CV (%)	Kurtosis	Skewness
Lutein	1.35	32.08	30.73	11.79	5.93	50.36	1.79	1.08
Zeax	0.02	2.90	2.88	0.49	0.51	107.38	4.71	1.23
β-car	0.04	2.29	2.25	0.52	0.35	67.07	3.27	1.11
Totcar	1.35	33.09	31.74	12.04	6.27	52.08	2.90	1.05
chl-a	1.10	66.07	64.97	5.06	8.45	139.12	6.86	2.44
chl-b	0.36	22.53	22.17	1.58	2.40	152.81	7.16	3.76
Totchl	0.36	87.68	87.32	4.05	8.52	213.26	9.96	3.03

Totcar, total carotenoid; Totchl, total chlorophyll; SD, standard deviation; CV, coefficient of variation.

**Table 2:** Soybean accessions identified with lutein and total carotenoids higher than 27 µg g^−1.^

ID	MG	Seed Coat Color	Country of Origin	Lutein (µg g^−1^)	Total Carotenoid (µg g^−1^) ^§^
WDD02708	I	brown	Russia	27.56	28.82
WDD02989	0	black	USA	28.13	29.62
ZDD08013	V	black	China	28.45	30.08
ZDD10734	VI	black	China	28.71	30.52
WDD02957	I	black	Russia	29.04	32.32
ZDD06375	IV	black	China	29.58	31.37
ZDD10248	VI	green	China	29.79	31.93
P1438498	IV	black	USA	29.93	31.59
WDD00475	IV	black	USA	30.54	31.95
ZDD11183	V	brown	China	32.08	33.09

ID, identification; MG, maturity group; ^§^ Total Carotenoid = sum of lutein, zeaxanthin and β-carotene mean contents.

## Data Availability

The data presented in this study are available in the article and Supplementary Materials.

## Supplementary Materials

The following are available online at https://www.mdpi.com/article/10.3390/plants11070848/s1, Figure S1: Seed samples of black (A); brown (B); yellow (C); green (D) seed coat colors of soybean seed germplasm accessions; Figure S2: The 100-seed weight of soybean accessions with various seed coat colors; Figure S3: The zeaxanthin, β-carotene, chlorophyll-a and -b concentrations of yellow-seed-coat-colored soybean accessions from different maturity groups; Table S1: The 408 soybean accessions of various origins along with their seed coat color and maturity group; Table S2: Monthly temperature and precipitation readings at the experimental sites in China in 2017 and 2018 cropping seasons; Table S3: Analysis of variance (ANOVA) for the effects of country of origin, maturity group, and seed color on carotenoid and chlorophyll concentrations of soybean germplasm accessions grown in China for two years; Table S4: Linear regression equation and R-squared values for the regression analysis between 100-seed weight and contents of lutein, zeaxanthin, β-carotene, total carotenoids, chlorophyll-a, chlorophyll-b and total chlorophylls from diversified soybean germplasm accessions with black (*n* = 41), brown (*n* = 18), green (*n* = 18) and yellow (*n* = 331) seed coat colors.
