# Supplementary material for: Origin, Maturity Group and Seed Coat Color Influence Carotenoid and Chlorophyll Concentrations in Soybean Seeds
Source: Plants (Basel). 2022 Mar 23;11(7):848. doi: 10.3390/plants11070848 (PMC9003432; doi:10.3390/plants11070848)
Supplement: Supplementary file 1 [file plants-11-00848-s001.zip › Figure S1. Various seed coat colors.pdf]

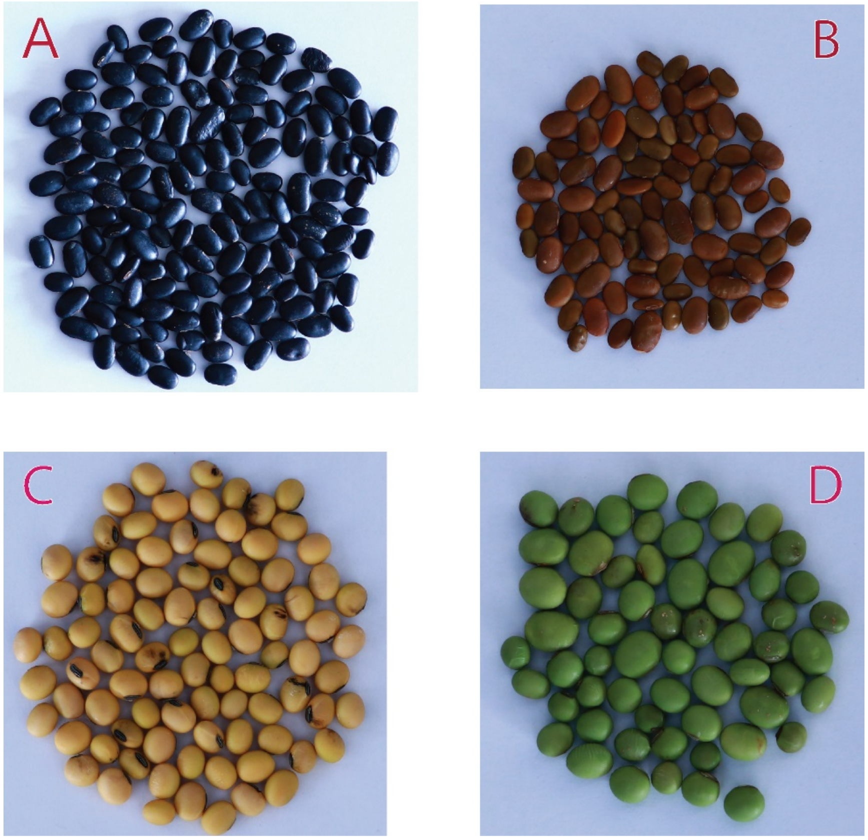

Figure S1. Seed samples of black (A); brown (B); yellow (C); and green (D) seed coat colors of soybean seed germplasm accessions.
