# Supplementary material for: Origin, Maturity Group and Seed Coat Color Influence Carotenoid and Chlorophyll Concentrations in Soybean Seeds
Source: Plants (Basel). 2022 Mar 23;11(7):848. doi: 10.3390/plants11070848 (PMC9003432; doi:10.3390/plants11070848)
Supplement: Supplementary file 1 [file plants-11-00848-s001.zip › Figure S2. The 100 Seeds dry weight of colored soybeans.pdf]

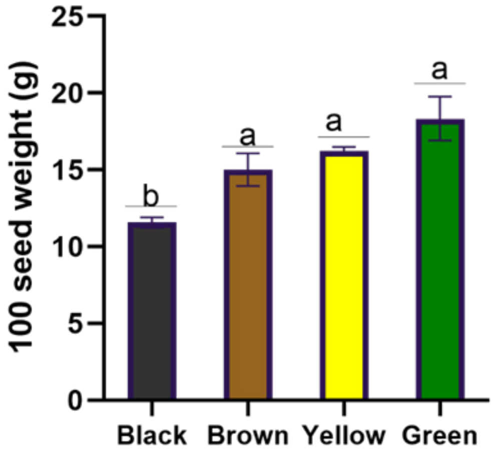

Figure S2. The 100 seeds weight of soybean accessions with various seed coat colors. Different lower-case letters indicate statistically significant difference at $p < 0.05$ level among the seed coat colors
