# Supplementary material for: Origin, Maturity Group and Seed Coat Color Influence Carotenoid and Chlorophyll Concentrations in Soybean Seeds
Source: Plants (Basel). 2022 Mar 23;11(7):848. doi: 10.3390/plants11070848 (PMC9003432; doi:10.3390/plants11070848)
Supplement: Supplementary file 1 [file plants-11-00848-s001.zip › Figure S3. Seed carotenoid and chlorophyll concentrations of soybean accessions from different maturity groups.pdf]

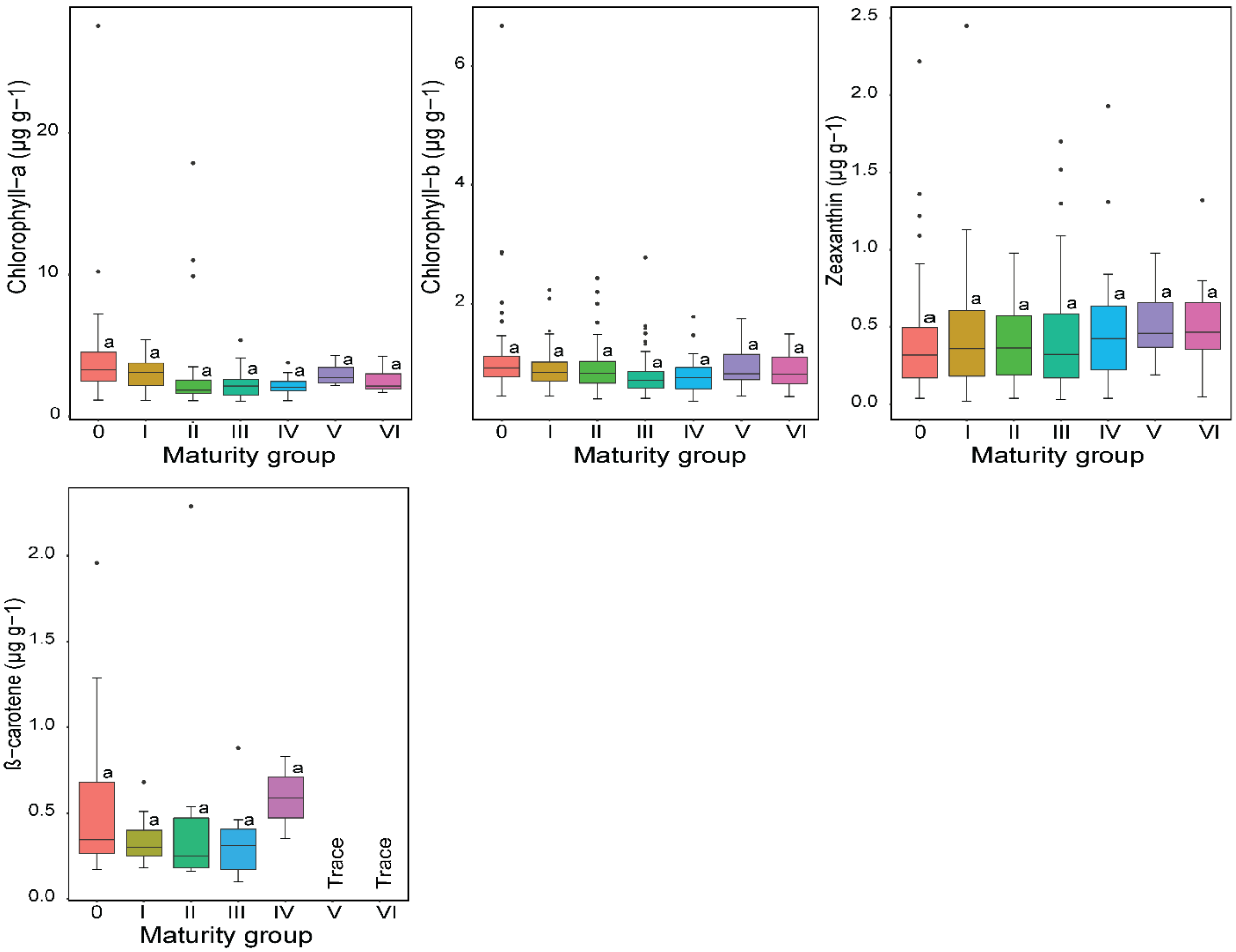

Figure S3. The zeaxanthin, β-carotene, chlorophyll-a &-b concentrations of yellow seed coat colored soybean accessions from different maturity groups. Similar lower-case letters indicate that there was non-significant difference ($p > 0.05$) among the maturity groups in each trait.
