# Supplementary material for: Origin, Maturity Group and Seed Coat Color Influence Carotenoid and Chlorophyll Concentrations in Soybean Seeds
Source: Plants (Basel). 2022 Mar 23;11(7):848. doi: 10.3390/plants11070848 (PMC9003432; doi:10.3390/plants11070848)
Supplement: Supplementary file 1 [file plants-11-00848-s001.zip › Supplementary Table S4. Regression analysis.pdf]

Table S4. Slopes, intercept coefficients and significance of regression equations between 100 seeds dry weight and contents of lutein, zeaxanthin, β-carotene, total carotenoids, chlorophyll-a, chlorophyll-b and total chlorophylls from diversified soybean germplasm accessions with black (n = 41), brown (n = 18), green (n = 18) and yellow (n = 331) seed coat colors

Seed coat color	Factor	Carotenoid components				Chlorophyll components		
		Lutein	Zeaxanthin	β-carotene	Total carotenoids	Chlorophyll-a	Chlorophyll-b	Total chlorophylls
Black	Slope	−1.0290	−0.0767	−0.0173	−1.1237	0.1284	−0.1281	−0.0052
	Intercept	30.6140	1.6453	0.8099	32.6806	9.6778	6.2746	15.5823
	P−value	**	**	NS	**	NS	NS	NS
Brown	Slope	−0.4959	−0.0242	−0.0443	−0.5896	−0.3187	−0.1725	−0.5042
	Intercept	19.4264	1.0398	0.8557	21.1902	9.7090	4.6684	12.6712
	P−value	NS	NS	*	NS	NS	*	*
Green	Slope	−0.1470	0.0267	−0.0104	−0.1523	−0.1397	−0.0771	−0.2355
	Intercept	15.8542	1.0412	0.6565	16.2128	10.9258	4.9030	14.9041
	P−value	NS	NS	NS	NS	NS	NS	NS
Yellow	Slope	−0.0431	−0.0034	−0.0118	0.0446	0.1724	0.0047	0.0350
	Intercept	11.2062	0.4965	0.6676	11.3786	0.5532	0.8437	1.2851
	P−value	NS	NS	NS	NS	**	NS	NS

*, ** significant at $p < 0.05$ and $p < 0.01$, respectively; NS, not significant.
