# Supplementary material for: Origin, Maturity Group and Seed Coat Color Influence Carotenoid and Chlorophyll Concentrations in Soybean Seeds
Source: Plants (Basel). 2022 Mar 23;11(7):848. doi: 10.3390/plants11070848 (PMC9003432; doi:10.3390/plants11070848)
Supplement: Supplementary file 1 [file plants-11-00848-s001.zip › Table S2-S3. Weather data at the Experimental Sites in China in 2017 and 2018 Cropping Seasons, and Aanalysis of Variance for Carotenoids and Chlorophyll contents.pdf]

Table S2. Monthly temperature, precipitation and sunshine readings at the experimental sites in China in 2017 and 2018 cropping seasons

**Sanya, Hainan Province**														
**Month**	**October**		**November**		**December**		**January**		**February**		**March**		**Sum**	
**Year**	**2017**	**2018**	**2017**	**2018**	**2017**	**2018**	**2017**	**2018**	**2017**	**2018**	**2017**	**2018**	**2017**	**2018**
Max. (°C)	29.0	28.0	28.0	24.0	25.0	22.0	24.0	23.0	24.0	24.0	27.0	34.0	157	155
Aver. (°C)	28.0	24.0	26.0	21.0	23.0	18.0	22.0	19.0	22.0	19.0	25.0	28.0	146	129
Min. (°C)	25.0	21.0	23.0	20.0	20.0	16.0	19	16.0	19.0	15.0	22.0	24.0	128	112
Rainfall (mm)	262.7	95.4	79.3	79.8	21.8	18.6	40.1	16.2	33.6	16.0	124.9	22.3	562.4	248.3
Sunshine (h)	166.5	202.5	136	90.5	132.5	155.5	131	124.5	138	173.5	200	245	904	991.5
**Changping, Beijing**														
**Month**	**June**		**July**		**August**		**September**		**October**		**November**		**Sum**	
**Year**	**2017**	**2018**	**2017**	**2018**	**2017**	**2018**	**2017**	**2018**	**2017**	**2018**	**2017**	**2018**	**2017**	**2018**
Max. (°C)	30.0	33.0	37.0	33.0	36.0	32.0	32.0	26.0	20.0	21.0	11.0	14.0	166	159
Aver. (°C)	28.0	28.0	35.0	29.0	34.0	28.0	29.0	21.0	18.0	19.0	9.0	11.0	153	136
Min. (°C)	23.0	22.0	31.0	25.0	30.0	24.0	24.0	17.0	15.0	15.0	6.0	8.0	129	111
Rainfall (mm)	67.0	35.5	69.5	127	135.8	57.2	8.2	19.0	84.4	3.7	0.0	2.7	364.9	245.1
Sunshine (h)	323.5	350.5	336	303	314	329	325	316	173.5	219	215	207	1687	1724.5

Table S3. Analysis of variance (ANOVA) for effect of country of origin, maturity group, and seed color on variability of carotenoid and chlorophyll concentrations in soybean germplasm accessions grown in China for two years

Sources of variation	Chlorophyll components			Carotenoid components			
	Chlorophyll-a	Chlorophyll-b	Totchl	Lutein	Zeaxanthin	β-carotene	Totcar
Accession	***	***	***	***	*	***	***
Year	***	***	NS	***	***	NS	***
Year×Accession	NS	NS	NS	NS	NS	NS	NS
Country	*	**	*	***	NS	*	**
Year	***	NS	NS	***	***	NS	***
Year × Country	NS	NS	NS	NS	NS	NS	NS
MG	NS	NS	***	***	NS	NS	***
Year	NS	***	***	***	***	NS	***
Year × MG	NS	NS	***	***	NS	NS	***
Seed color	***	***	***	***	***	***	***
Year	***	***	NS	***	***	NS	***
Year × Seed color	NS	NS	NS	***	***	NS	***

*, ** and *** represent the significance levels at $p < 0.05$, 0.01, and 0.001, respectively; NS, not significant; Totchl, total chlorophyll; Totcar, Total carotenoid; MG, Maturity group.
